# A modified standard American diet induces physiological parameters associated with metabolic syndrome in C57BL/6J mice

**DOI:** 10.3389/fnut.2022.929446

**Published:** 2022-08-29

**Authors:** Sophie B. Chehade, George B. H. Green, Christopher D. Graham, Ayanabha Chakraborti, Bijal Vashai, Amber Moon, Michael B. Williams, Benjamin Vickers, Taylor Berryhill, William Van Der Pol, Landon Wilson, Mickie L. Powell, Daniel L. Smith, Stephen Barnes, Casey Morrow, M. Shahid Mukhtar, Gregory D. Kennedy, James A. Bibb, Stephen A. Watts

**Affiliations:** ^1^Department of Biology, University of Alabama at Birmingham, Birmingham, AL, United States; ^2^Department of Surgery, University of Alabama at Birmingham, Birmingham, AL, United States; ^3^Comprehensive Cancer Center, University of Alabama at Birmingham, Birmingham, AL, United States; ^4^Center for Clinical and Translational Science, University of Alabama at Birmingham, Birmingham, AL, United States; ^5^Department of Pharmacology and Toxicology, University of Alabama at Birmingham, Birmingham, AL, United States; ^6^Department of Nutrition Sciences, Nutrition Obesity Research Center, University of Alabama at Birmingham, Birmingham, AL, United States; ^7^Department of Cell, Developmental, and Integrative Biology, University of Alabama at Birmingham, Birmingham, AL, United States

**Keywords:** obesity, metabolic syndrome, Western Diet, high fructose corn syrup, high fat diet

## Abstract

Investigations into the causative role that western dietary patterns have on obesity and disease pathogenesis have speculated that quality and quantity of dietary fats and/or carbohydrates have a predictive role in the development of these disorders. Standard reference diets such as the AIN-93 rodent diet have historically been used to promote animal health and reduce variation of results across experiments, rather than model modern human dietary habits or nutrition-related pathologies. In rodents high-fat diets (HFDs) became a classic tool to investigate diet-induced obesity (DIO). These murine diets often relied on a single fat source with the most DIO consistent HFDs containing levels of fat up to 45-60% (kcal), higher than the reported human intake of 33–35% (kcal). More recently, researchers are formulating experimental animal (pre-clinical) diets that reflect mean human macro- and micronutrient consumption levels described by the National Health and Nutrition Examination Survey (NHANES). These diets attempt to integrate relevant ingredient sources and levels of nutrients; however, they most often fail to include high-fructose corn syrup (HFCS) as a source of dietary carbohydrate. We have formulated a modified Standard American Diet (mSAD) that incorporates relevant levels and sources of nutrient classes, including dietary HFCS, to assess the basal physiologies associated with mSAD consumption. Mice proffered the mSAD for 15 weeks displayed a phenotype consistent with metabolic syndrome, exhibiting increased adiposity, fasting hyperglycemia with impaired glucose and insulin tolerance. Metabolic alterations were evidenced at the tissue level as crown-like structures (CLS) in adipose tissue and fatty acid deposition in the liver, and targeted 16S rRNA metagenomics revealed microbial compositional shifts between dietary groups. This study suggests diet quality significantly affects metabolic homeostasis, emphasizing the importance of developing relevant pre-clinical diets to investigate chronic diseases highly impacted by western dietary consumption patterns.

## Introduction

Obesity and related metabolic syndrome (MetS) are widespread in developed countries. Dietary consumption patterns generally referred to as the “Western Diet” (WD) have been scrutinized as possible causes of MetS (1, 2). The WD is characterized by increased consumption of processed foods, added sugars, saturated fat, sodium, and a high ratio of omega-6: omega-3 polyunsaturated fatty acids (PUFAs). The WD pattern is typically low in fibrous fruits and vegetables (3). From 1970 to 2003, the average daily calorie intake increased by 523 kcal with a concomitant 63% increase in consumption of animal fats and vegetable oils as well as a 19% increase of sugars (4). The quantity and quality of these dietary fats and sugars have been investigated as possible causes of diet-related health disorders (5, 6).

Understanding the etiology of MetS is paramount to identifying and treating at-risk populations. The strong association between WD patterns and the development of MetS highlights the importance of establishing a pre-clinical model that incorporates human-relevant dietary patterns (2). Currently, most researchers applying pre-clinical rodent models utilize standardized reference diets, such as AIN-93, in their studies (3). These semi-purified diets were originally created to address concerns of variation in results among experiments and are formulated to promote reproducible health and reproductive parameters of the model organism (7). Although these diets have been invaluable for standardizing murine research (8), they may not be adequate to model human nutrition-related pathologies. Historically, nutrition experiments utilizing rodent models employ experimental diets that are produced by modification of reference diets for specific ingredients or nutrient classes, such as protein, fat, or carbohydrate. Although useful for understanding disease pathologies induced by one specific nutrient change (over or under exposure), these altered diets are not representative of what is being consumed on average in the United States and have limited translatability to the average human population (2). For example, the classic high-fat diet (HFD) contains approximately 45–60% of kcal as fat derived from one or few dietary fat sources, typically lard and/or tallow, and has been successful in inducing MetS and associated pathologies (9). According to the CDC, however, the mean fat intake for men and women over 20 years old is approximately 34–35% of their caloric intake (10). Similar to the pitfalls of HFDs, choline-deficient diets are utilized to induce non-alcoholic fatty liver disease (NAFLD) associated with MetS, yet they fail to produce NAFLD that manifests in a physiologically relevant way to the human population (2). While of basic interest, these commonly used experimental diets may not accurately replicate the pathogenesis of metabolic disorders, resulting in poor translatability to human populations.

Recently, two groups of investigators designed WDs in accordance with survey responses reported by the National Health and Nutrition Examination Survey (NHANES) to reflect average human consumption patterns. Monsanto et aldeveloped the Total Western Diet (TWD) to address overestimations of fat intake as well as lack of inclusion of multiple sources of fat in previously established diets (3). Totsch et al. further modified the TWD to produce the Standard American Diet (SAD), incorporating multiple sources of dietary fat as well as human-relevant levels of omega-6: omega-3 PUFA (11). The primary source of carbohydrate in both the TWD and the SAD is sucrose; however, NHANES reports that sugar-sweetened beverages (SSBs) are primary contributors to added sugars being consumed across multiple age-groups in the United States (12). SSBs such as soft drinks are commonly sweetened with high fructose corn syrup (HFCS) and are associated with an increased risk for obesity (13).

There is increasing evidence that fructose consumption may be related to the development of metabolic disorders associated with obesity (2, 14–18). Following its introduction in the 1970s, HFCS consumption markedly increased until the early 2000s (19). During this time, rates of obesity increased (20), leading many to hypothesize that fructose may be at least partially responsible (21). The HFCS contained in SSBs, HFCS-55, is an unequal mixture of glucose and fructose monosaccharides that are produced by a series of enzymatic reactions which convert extracted corn starch to glucose and fructose at levels of ca. 45 and 55%, respectively (22). In contrast, sucrose is a disaccharide composed of a 50:50 ratio of glucose: fructose.

To investigate the association of WD and the development of MetS, we have formulated a modified version of the SAD that reflects above-average macronutrient intakes as reported by NHANES. The modified SAD (mSAD) incorporates HFCS within the feed, an important distinction from diets that introduce HFCS in an *ad libitum* drinking solution (23). Beyond formulating the diet, we have characterized the basal physiologies of C57BL/6J mice consuming this diet. We hypothesize that a diet that represents above-average human intakes will produce metabolic phenotypes consistent with those seen in human populations with a greater incidence of obesity and MetS.

## Materials and methods

### Animals

All experiments were approved by the Institutional Animal Care and Use Committee at the University of Alabama at Birmingham (UAB), Birmingham, AL (IACUC-21005). Male C57BL/6J mice were obtained from Jackson Labs (000664) at 4 weeks of age and housed in AAALAC-approved facilities at UAB with a 12:12 h dark: light cycle (06:00–18:00). Mice were housed in groups of 3 in wire-top cages with *ad libitum* access to food and sterile water. Mice were acclimated for 7 days and then randomly assigned to the AIN93M control diet (CON) or the experimental diet (mSAD) at 5 weeks of age. Mice were proffered diets for 15 weeks. Food and water intake for each cage of mice were recorded weekly, and nutrient intake parameters were calculated from these records.

### Diet

C57BL/6J male mice were assigned randomly to one of two groups (*n* = 12 total, *n* = 6/group, *n* = 3/cage, *n* = 2 cages/group) and proffered either the CON or the mSAD for a period of 15 weeks. The CON (AIN-93M, TD.94048, Envigo, Madison, WI, United States) is formulated for optimal health and is a common standard reference diet used in rodent research (7). AIN-93M consists of 12.4% protein (13.7% kcal), 68.3% carbohydrate (75.9% kcal), and 4.1% fat (10.3% kcal) (Table 1). The modified Standard American diet (mSAD, TD.180061, Envigo, Madison, WI, United States) was formulated to approximate 50th percentile macronutrient intakes from data reported by NHANES. Macro- and micronutrient levels are listed in Table 1 and Table 2, respectively. The mSAD is composed of 12.3% protein dry matter (12.2% kcal), 49.5% carbohydrate (49.2% kcal), and 17.3% fat (38.6% kcal) (Table 1). The mSAD contains HFCS as the primary source of carbohydrate at a level of 26% of the diet (by weight). Multiple sources of saturated and unsaturated fat were incorporated in the mSAD, including palm oil, soybean oil, corn oil, cottonseed oil, lard, beef tallow, and anhydrous milkfat (Table 1). Moreover, the mSAD contains reduced levels of fiber and increased levels of sodium, which are supported by NHANES data (7).

**TABLE 1:** AIN-93M control diet (CON) and modified Standard American Diet (mSAD) formulations.

AIN-93M		mSAD	
Ingredient	g/kg	Ingredient	g/kg
Casein	140	Casein	83
L-cysteine	1.8	L-cysteine	1.8
Corn starch	465.692	Whole wheat flour	374.77
Maltodextrin	155	High fructose corn syrup	264
Sucrose	100	Maltodextrin	60
Soybean oil	40	Palm oil	59.5
Cellulose	50	Soybean oil	17.3
Mineral mix	35	Corn oil	10.6
Vitamin mix	10	Cottonseed oil	20.9
Choline bitartrate	2.5	Lard	18
TBHQ, antioxidant	0.008	Beef tallow	16
		Anhydrous milkfat	23.3
		Cholesterol	0.4
		Sodium chloride	4.0
		Mineral Mix	35
		Vitamin Mix	10
		Choline bitartrate	1.4
		TBHQ, antioxidant	0.03
**Macronutrient**	**% kcal**	**Macronutrient**	**% kcal**
Protein	13.7	Protein	12.2
Fat	10.3	Fat	49.2
Carbohydrate	75.9	Carbohydrate	38.6
**Kcal/g 3.6**		**Kcal/g 4.0**	
**Other Nutrients**	**%**	**Other Nutrients**	**%**
Fiber*	5	Fiber*	3.75
Moisture	9.87	Moisture	10.4

*Fiber levels approximated by the addition of individual ingredient levels.

**TABLE 2:** AIN-93M control diet (CON) and modified Standard American Diet (mSAD) micronutrient profiles.

	AIN-93M	mSAD
**Vitamins**		
Vitamin A (IU/kg)	4000	3,951
Vitamin D (IU/kg)	1000	400
Vitamin E (IU/kg)	75	28
Vitamin K (mg/kg)	0.8	0.2
Biotin (mg/kg)	0.2	0.2
Choline (mg/kg)	1147.5	755
Folic Acid (mg/kg)	2	1.4
Niacin (mg/kg)	30	69.1
Pantothenate (mg/kg)	14.7	17
Riboflavin (mg/kg)	6	5
Thiamin (mg/kg)	4.9	5
Vitamin B6 (mg/kg)	5.8	5.4
Vitamin B12 (mg/kg)	0.03	0.01
**Minerals**		
Calcium (g/kg)	5	2.1
Phosphorus (g/kg)	3	3.3
Potassium (g/kg)	3.6	6.6
Sodium (g/kg)	1	7
Chlorine (g/kg)	1.6	10.8
Magnesium (g/kg)	0.5	1.1
Copper (mg/kg)	6.1	4.2
Iron (mg/kg)	36.9	45
Zinc (mg/kg)	39.8	36.8
Manganese (mg/kg)	10.5	25.5
Iodine (mg/kg)	0.21	0.21
Selenium (mg/kg)	0.15	0.44
Molybdenum (mg/kg)	0.15	0.15
Chromium (mg/kg)	1	1

### Growth metrics and body composition

To assess body weight over time, individual body weights were measured weekly. Weight gain was calculated by subtracting the previous week’s weight from the current week. Specific growth rate (SGR) was calculated using the following formula:

S⁢G⁢R=[(w⁢e⁢e⁢k⁢ 2⁢w⁢e⁢i⁢g⁢h⁢tw⁢e⁢e⁢k⁢ 1⁢w⁢e⁢i⁢g⁢h⁢t)1Δ⁢t-1]*100%.

Quantitative Magnetic Resonance (QMR) was performed according to previously published methods (24) on mice at weeks 4, 6, 7, 10, and 12 to measure *in vivo* fat and lean-tissue mass (EchoMRI™ 3-in-1; Echo Medical Systems, Houston, TX, United States). Due to technical issues, QMR could not be performed at the termination of the study; however, QMR lean mass values for week 15 were predicted using a simple linear regression.

### Glucose tolerance and insulin tolerance testing

Glucose tolerance tests (GTT) and insulin tolerance tests (ITT) were performed to evaluate glucose and insulin response, respectively. Tests were performed after 4, 6, 10, and 12 weeks of diet exposure. Mice were weighed and fasted prior to testing (5-h fast for GTT, 4-h fast for ITT). For GTTs, mice received i.p. injection of a 25% glucose solution in phosphate-buffered saline (PBS) at a concentration of 2 g/kg body weight and blood glucose was monitored at 0, 15, 30, 60, and 120 min post-injection. For ITTs, mice received i.p. injection of insulin at 0.5 U/kg body weight. Blood glucose was measured at 0, 15, 30, 45, 60, and 120 min following injection. All blood samples were collected via tail vein venipuncture and blood glucose was measured using a Contour glucose meter (Bayer 82486543).

### Tissue collection and analysis

Following termination of the study, mice were euthanized by decapitation and trunk blood was collected for downstream analyses. Adipose depots (inguinal, dorsal, peri-renal, gonadal, mesenteric, and interscapular) and internal organs (kidney, liver, spleen, and alimentary canal) were dissected and weighed individually. Following overnight fixation in 10% Neutral Buffered Formalin (NBF), liver and adipose tissues were processed and embedded in paraffin by UAB’s Molecular Detection Core Facility. Tissues were sectioned at 5 μm and mounted onto glass slides. To assess hepatic lipid deposition, liver sections were stained with Hematoxylin and Eosin (H&E). For CLS detection, adipose sections were stained with anti-Mac-2 antibody (1:2,800; Cedarlane Laboratories 61R-1589) followed by hematoxylin counterstaining. Slides were imaged using a Nikon stereomicroscope (Nikon SMZ1000N, Nikon, Melville, NY, United States). The total area occupied by CLS from the full field of each tissue section was quantified using NIS Elements imaging software (NIS Elements BR 3.2, Nikon, Melville, NY, United States).

### Quantitative PCR

Total RNA was collected from proximal colon tissue using the RNeasy Plus Mini Kit (Qiagen 74134) per the manufacturer’s instructions followed by quantification and purity assessment on a NanoDrop One (Thermo Fisher Scientific). RNA with a concentration >30 ng/μl and A260/280 > 2.00 was processed into complementary DNA (cDNA) using a High Capacity cDNA Reverse Transcription Kit (Applied Biosystems 4368814) per the manufacturer’s instructions (200 ng RNA/reaction). cDNA was used in duplicate as a template for qRT-PCR using Taqman Fast Advanced Master Mix (Applied Biosystems 4444556) and the following primers on a QuantStudio 6 Flex (Applied Biosystems): Actin (Mm02619580_g1), TNFα (Mm00443258_m1), IL-1β (Mm00434228_m1), IL-6 (Mm00446190_m1). The 2^(-delta Ct) method was used to calculate relative gene expression from raw cycle threshold (Ct) values and target gene expression was normalized to actin.

### Microbiome analysis

Microbiome analysis was performed according to previously published methods (25). After 15 weeks of diet, feces were collected and fecal DNA was isolated using the Fecal DNA Isolation Kit (Zymo Research, Irvine, CA, United States, cat. no. 06010) and a 250 base pair amplicon library was generated via PCR with primers specific for the V4 region of the rRNA gene. PCR products were electrophoresed on agarose gel and subsequently excised from the gel and purified using the QIAquick Gel Extraction Kit (Qiagen, cat. no. 28704). Next-generation sequencing was accomplished using the Illumina MiSeq (Illumina, San Diego, CA, United States, cat. no. SY-410-1003) and microbiome analysis was performed using QIIME2 (Quantitative Insights Into Microbial Ecology) (25).

### Taxonomic distribution

The taxonomic composition of the mouse fecal mSAD and control samples were determined utilizing QIIME2 (v.2021.11) bioinformatics tools (26). The samples raw sequence files was demultiplexed, via the Casava 1.8 paired-end demultiplexed fastq format, followed by denoising using DADA2 (q2-dada2) for quality filtering (27). The alignment of amplicon sequence variants (ASVs) was achieved using mafft (q2-alignment) (28), and the results were exported into fasttree2 (q2-phylogeny plugin) for phylogeny construction, using the FastTree building method (default setting) (29). The “Core-metrics-phylogenetic” command (q2-diversity plugin) generated Alpha-diversity metrics (Faith’s Phylogenetic Diversity, and observed features) (30), Shannon (31), Simpson (32), chao1 (33), beta diversity metrics, un-weighted UniFrac, weighted UniFrac (34), Principle Coordinate Analysis (PCoA), Bray–Curtis dissimilarity, and Jaccard distance. The samples based off the minimum value of 15,411 sequences per sample were rarefied (subsampled without replacement). Taxonomic assignment to ASVs was achieved via q2-feature-classifier plugin (35), utilizing the “classify-sklearn” command against the silva-138-99-nb-classifier (36). The taxa were then collapsed into table format using the “qiime taxa collapse” command. Adonis and PERMANOVA beta-diversity metrics were determined via “qiime diversity beta-group-significance” plug-in (37).

### Statistics

Statistical analysis was performed using Prism 9.0 software (GraphPad Software, San Diego, CA, United States) and R software (R Core Team (38)). Statistical significance was determined using Student’s *t*-test (when comparing two groups), Welch’s T-test (when comparing two groups with unequal variances), Wilcoxon T-test (when comparing two groups when data was not normally distributed), one-way analysis of variance (ANOVA) (when comparing more than two groups with one independent variable) or two-way ANOVA (when comparing more than two groups with two or more independent variables). When applicable, a Tukey–Kramer post-hoc test was used to calculate the Minimum Significant Difference (MSD) between any pair of means and statistical significance was assigned to groups with differences between the means greater than the MSD. A *p*-value < 0.05 was considered significant.

## Results

**FIGURE 1:**
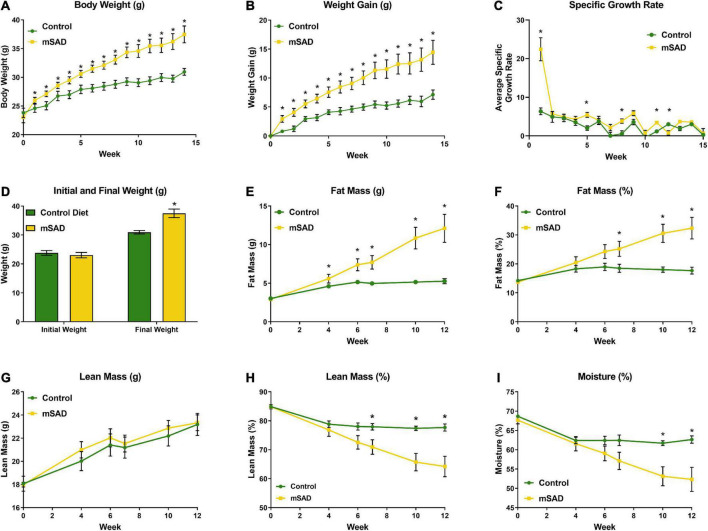
Effects of diet on weight and body composition. Male C57BL/6J mice were proffered the CON or the mSAD diet for a 15-week period. **(A)** Body weight was measured weekly (*n* = 12/diet). **(B)** Cumulative weight gain and **(C)** specific growth rate for each diet. **(D)** Initial and final weights for each diet. QMR analysis at 4, 6, 7, 10, and 12 weeks (*n* = 6/diet) includes **(E)** g fat mass, **(F)** % fat mass, **(G)** g lean mass, **(H)** % lean mass, and **(I)** percent water. Values are represented as mean ± SEM. Data were compared using Welch’s *t*-test. *p < 0.05.

Weight and body composition parameters recorded during the study revealed immediate and significant changes to the physical state of mice in response to mSAD feeding. After 15 weeks, mSAD-fed mice exhibited significantly more weight gain and a higher SGR than their CON counterparts (Figures 1A–D). Mice fed the mSAD accumulated higher absolute amounts of fat mass as well as a percentage of body weight than CON (Figures 1E,F). Both diets induced lean mass accumulation at a similar rate throughout the study (Figure 1G), but not as a percentage of total mass (Figure 1H).

**FIGURE 2:**
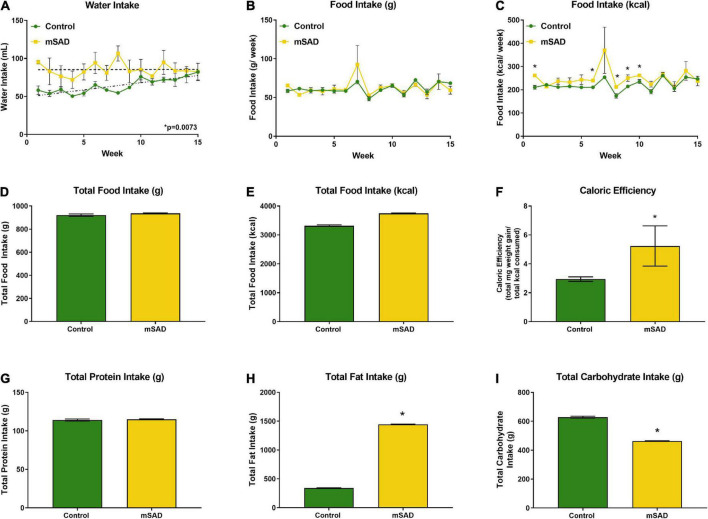
Water, food, and macronutrient intake. All values represent average cage intake (*n* = two cages, each containing three mice). Water intake was measured as mL per cage per week **(A)**. Food intake was measured as g per cage per week by a subtractive method of weekly provision minus weekly remnants **(B)** and kcal consumed per week **(C)**. Total food intake (g) **(D)** and kcal consumption **(E)** for the 15-week period. Caloric efficiency for each diet was calculated as total mg weight gain/total kcal consumed for the 15-week period **(F)**. Total protein **(G)**, lipid **(H)**, and carbohydrate intake **(I)** were calculated. Values are represented as mean ± SEM. Data were compared using Welch’s *t*-test. *p < 0.05.

Within the first week, water intake was higher in the mSAD diet compared to CON (Figure 2A). However, water intake in CON mice increased over the feeding period. Water intake values displayed high levels of variance, in some cases due to issues with leaky sippers that were noted at a few timepoints during the study. No change in weekly or overall food intake was observed between diet treatments (Figures 2B–D); however, because the mSAD had a higher caloric density, total energy intake in mSAD-fed mice was slightly higher (3,742 kcal) than in CON (3,314 kcal) (Figure 2E). Caloric efficiency, defined as total mg of weight gained/total kcal consumed, was significantly higher in mSAD-fed mice (Figure 2F). At the macronutrient level, mSAD-fed mice consumed more fat and less carbohydrate than CON mice, with no differences in protein intake (Figures 2G–I).

**FIGURE 3:**
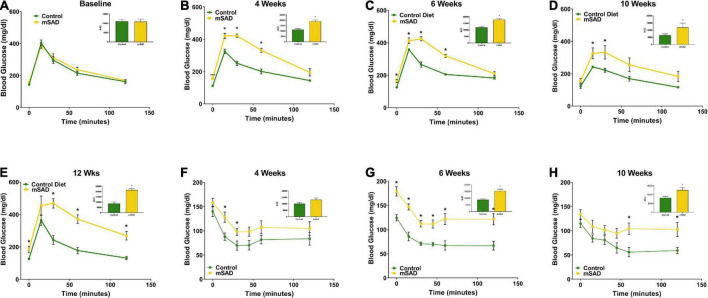
Effects of diet on glucose and insulin response. Glucose tolerance tests were performed prior to the feeding regimen **(A)** and after weeks 4 **(B)**, 6 **(C)**, 10 **(D)**, and 12 **(E)** weeks of diet. Insulin tolerance tests were performed after weeks 4, **(F)**, 6 **(G)**, and 10 **(H)** weeks of diet. Values are represented as mean ± SEM (*n* = 4–6/time point). Data were compared using Welch’s *t*-test. *p < 0.05.

To assess whether the changes in body composition in response to mSAD intake are associated with metabolic alterations in glucose and insulin signaling, GTT and ITT were recorded during the study period. Prior to diet administration, mice showed no difference in fasting blood glucose or glucose tolerance (Figure 3A). However, mSAD-fed mice displayed fasting hyperglycemia and impaired glucose tolerance at every timepoint measured relative to CON mice (Figures 3B–E). Similarly, mSAD-fed mice display impaired insulin responsiveness at all time points measured (Figures 3F–H). At week 12, 4 of 6 control mice fell below the glucose threshold, forcing premature termination of the assay with no subsequent data collection.

**FIGURE 4:**
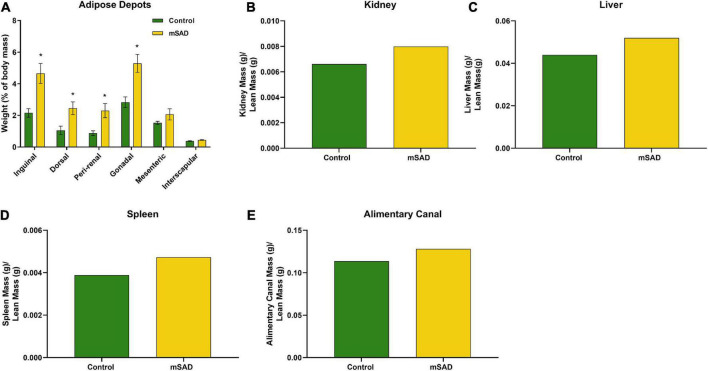
Effects of diet on an adipose depot and organ weights. Weights of the inguinal, dorsal, peri-renal, gonadal, mesenteric, and interscapular adipose depots were compared between CON- and mSAD-fed mice after 15 weeks of feeding **(A)**. Kidney **(B)**, liver **(C)**, spleen **(D)**, and alimentary canal **(E)** weights were also evaluated in both groups. Adipose depot values are represented as % of total body mass. Organ weight values are represented as absolute mass normalized by regression-predicted lean mass (g/g). When possible, data were compared using Student’s t-tests. *p < 0.05.

Following termination of the study, mice were euthanized, and adipose depots, kidneys, liver, spleen, and alimentary canal were dissected and weighed for further assessment of mSAD alterations at the tissue-level. Inguinal, dorsal, peri-renal, and gonadal depot weights of mSAD-fed mice were significantly heavier than CON mice (Figure 4A). Organ weights were evaluated by normalizing their absolute weight with regression-predicted lean matter weights at the 15-week timepoint. Although data cannot be statistically compared, organs of mSAD animals overall appeared larger than those in the control group (Figures 4B–E).

**FIGURE 5:**
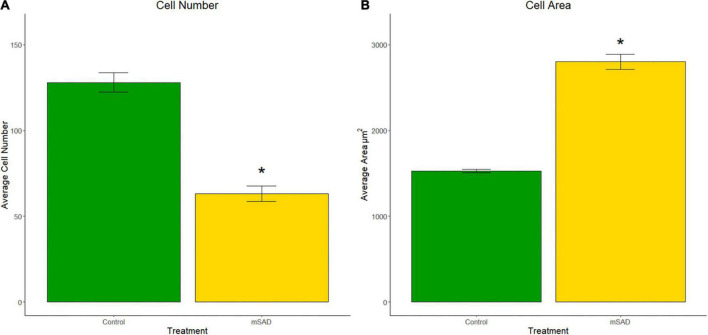
Effects of diet on gonadal adipocyte hyperplasia and hypertrophy. Cell counts and cell area measurements were performed on FFPE sections of gonadal fat pads stained with hematoxylin. Cell counts **(A)** and cell area measurements **(B)** were quantified. Values are representative of average cell count and average cell area (μm^2^) of multiple regions of interest (CON, *n* = 6; mSAD, *n* = 3) each measuring 500 μm × 500 μm. Data were compared using the unpaired Wilcoxon rank-sum test. *p < 0.001.

To understand cellular responses within the larger adipose mass with mSAD diets, adipocyte hyperplasia and hypertrophy were measured on gonadal tissue sections of CON- and mSAD-fed mice. Cell counts to evaluate adipocyte hyperplasia within equivalent areas of the tissue revealed significantly lower cell numbers in mSAD fat pads compared to CON (Figure 5A). Quantification of adipocyte cell area indicated a significantly larger average cell area in mSAD tissue sections compared to CON (Figure 5B).

**FIGURE 6:**
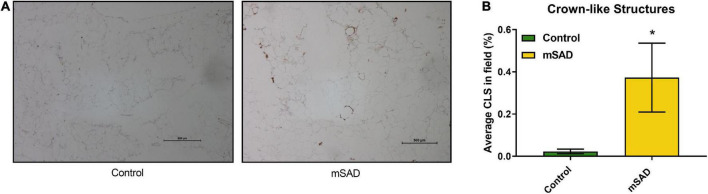
Effects of diet on gonadal fat pad inflammation. FFPE sections of gonadal fat pads from CON- or mSAD-fed mice were incubated with anti-MAC-2 followed by counter-staining with hematoxylin **(A)**. Quantification of CLS **(B)**. Values are represented as the average % area of CLS occupied in the total field of tissue sections. Data were compared using Student’s *t*-test. *p < 0.05.

The presence of CLS in gonadal adipose tissue sections of CON- and mSAD-fed mice was evaluated histologically (Figure 6A) followed by quantification of the percent area of CLS occupied in the total field of each tissue section (Figure 6B). mSAD-fed mice samples displayed a significantly higher percentage of CLS compared to CON mice.

**FIGURE 7:**
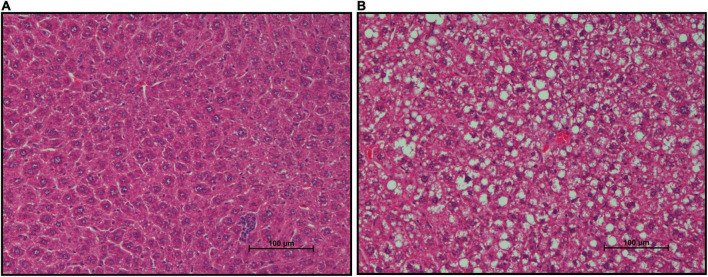
Effects of diet on hepatic lipid accumulation. Representative images of Formalin-fixed Paraffin-Embedded (FFPE) livers from six mice fed either CON diet with no lipid deposition **(A)** or mSAD with lipid deposition **(B)**. Tissue sections were stained with Hematoxylin and Eosin (H&E). Scale bars at the bottom right of images represent 100 μm.

FFPE liver sections of mice from both groups were stained with H&E to assess lipid deposition (Figures 7A,B). Gross increases in lipid deposits were observed in one of six CON mice and two of six mSAD-fed mice.

**FIGURE 8:**
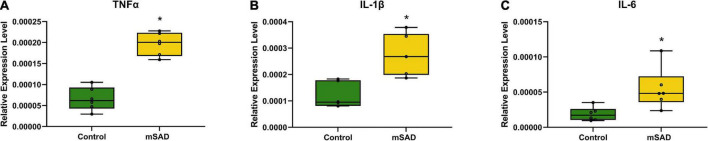
Effects of diet on colonic inflammation. Relative gene expression levels of *Tnf*α **(A)**, *Il-1*β **(B)**, and *Il-6* **(C)** in colon tissues of CON- and mSAD-fed mice. Values are represented as relative expression levels normalized to β-*actin*. Data were compared using one-way ANOVA. *p < 0.05.

The presence of CLS within adipose tissue and hepatic steatosis in mSAD animals suggested that pro-inflammatory processes associated with mSAD feeding may affect multiple organ systems. To evaluate the role of inflammation in the colon, pro-inflammatory cytokine levels were measured. mSAD-fed mice exhibited significantly elevated gene expression levels of Tumor Necrosis Factor α (Tnfα), Interleukin 1 β (Il-1β), and Interleukin 6 (Il-6) relative to CON mice (Figures 8A–C) in colon tissues.

**FIGURE 9:**
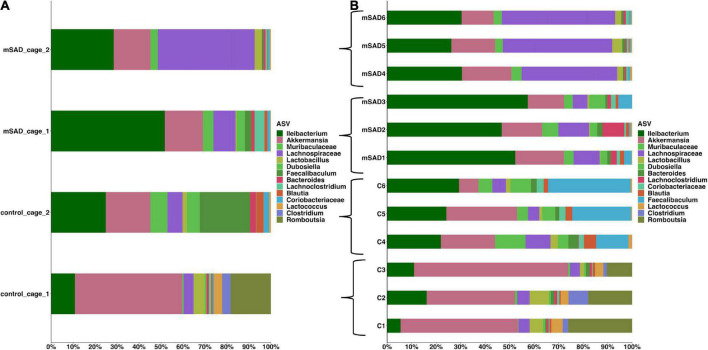
The mean of each sample group, according to cage was plotted for clarity, across sample groups **(A)**. Sample assignments are as follows: mSAD = mice fed the mSAD diet, control/con = mice feed the control diet, and cage represents their respective cage. Relative abundance stacked column bar graph showing top 15 taxa at the most resolved level across all samples (*n* = 3 for each sample) in the gut ecosystem of *mus musculus* **(B)**. Taxonomic identities were based on their assignment through the (SILVA v138) database as determined by the Quantitative Insights into Microbial Ecology (QIIME_2, v2021.11) and graphed using R (ggplot package).

**FIGURE 10:**
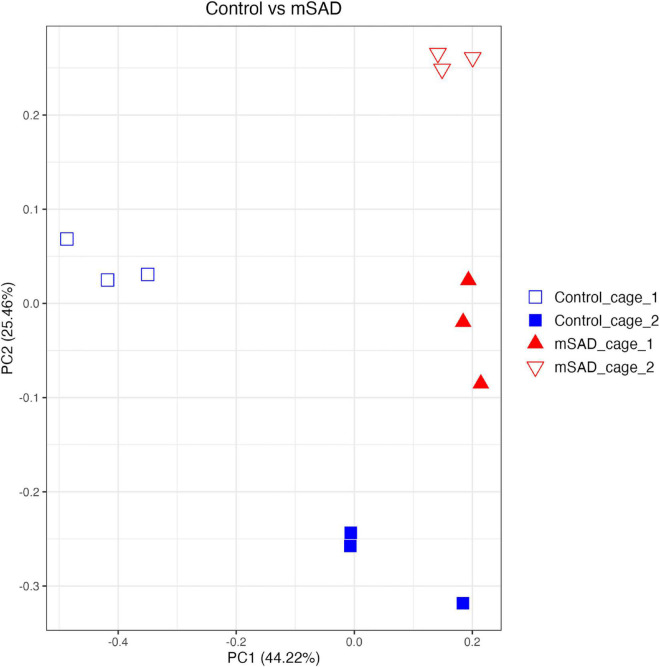
Beta diversity analysis of gut microbiota of mouse samples was observed across similarity metrics determined via QIIME2 (2021.11). A Bray–Curtis PCOA plot displays sample clustering patterns based on observed ASVs. plotted with R (ggplot package), and the q2-qiime diversity beta-group-significance. The group assignments are indicated as follows: control_cage_1 (blue open square; *n* = 3); control_cage_2 (blue square; *n* = 3); mSAD_cage_1 (red triangle; *n* = 3); mSAD_cage_2 (red open triangle; *n* = 3).

Increased levels of pro-inflammatory mediators prompted further analysis of the impact of the mSAD on the composition of the fecal microbe community. Using 16S rRNA sequencing and QIIME2 (v2021.11) analysis of pooled fecal samples, we assessed alpha and beta diversity metrics between CON and mSAD groups controlling for possible cage effects (Table 3 and Figures 9A,B, 10). Comparisons of alpha diversity in CON and mSAD samples measured by the Shannon index with cage input as a random effect produced a significant effect of diet (*p* = 0.04). Simpson and Chao1 comparisons with cage as a random effect produced nonsignificant results (*p* = 0.11, *p* = 0.32) (Table 3). Analysis of Bray-Curtis calculations (25) revealed significant differences between the microbe communities in the CON versus mSAD (BC < 0.001) indicating that exposure of mice to mSAD resulted in a re-structuring of the microbial community so that it was now distinct from that found in mice fed the CON diet. Visualization of changes in beta diversity of the sample groups can be seen in the principal coordinate analysis (PCOA) in Figure 10. Cluster separation seen in the PCOA plot between control cage 1 and control cage 2 was controlled for during analysis of beta diversity metrics with the Adonis test and resulted in a significant difference between diet groups (*p* < 0.001), and 83% (*R*^2^ = 0.826) of the variation seen is due to the diet (Supplementary Table 2). Further evaluation of microbial composition within control cage 1 and control cage 2 was explored to assess the slight separation of the two cages seen in Figure 10. This assessment revealed that differences between these cages may be primarily influenced by two genera of microbes. In cage 1, genus *Romboutsia* represents approximately 9–25% of the microbiome, while in cage 2, genus *Faecalibaculum* represents ∼10–30% of the microbiome. Across both cages and all six mice, all other genera are present in similar proportions (Supplementary Table 3). The pooled fecal samples of mSAD and CON fed mice groups revealed taxa assigned to the *Firmicutes* phylum to be most abundant across groups (Figure 9A). *Ileibacterium*, and *Akkermansia* to be most abundant across diet groups. *Ileibacterium* was found to the more dominant taxon in the mSAD group (∼22–50%), compared to the control group (∼5–28%). The control group revealed a larger abundance of *Akkermansia* (∼7–57%), in contrast to the mSAD group (∼11–17%). A unique abundance of Lachnospiraceae family members (∼5–40%) were observed in the mSAD group, in contrast to the CON group (∼4–9%). The CON diet group showed distinct abundances of *Faecalibaculum* (0–31%) and *Romboutsia* (0–24%).

**TABLE 3:** Alpha diversity metrics were determined via QIIME2 diversity alpha plugin (QIIME_2, v2021.11), clustered according to cage_1_con (samples C1:C3, *n* = 3), cage_2_con (samples C4:C6, *n* = 3), cage_1_ex (samples mSAD1:mSAD3, *n* = 3), cage_2_ex (samples mSAD4:mSAD6, *n* = 3).

Sample_Name	Cage	chao1	Shannon	Simpson	Faith pd
C1	cage_1_con	87	3.41916094	0.77558052	8.09726054
C2	cage_1_con	95	3.90299357	0.84905891	7.87877218
C3	cage_1_con	82	2.96225955	0.65589128	9.11090517
C4	cage_2_con	68	4.24052121	0.90009793	5.93279296
C5	cage_2_con	55	3.31761141	0.82603949	5.53401411
C6	cage_2_con	51	3.44943346	0.82690652	5.09134285
mSAD1	cage_1_ex	63	3.68744323	0.82162558	5.86502142
mSAD2	cage_1_ex	103	4.65994564	0.88047728	8.83570895
mSAD3	cage_1_ex	67	3.4966321	0.77587981	7.03250057
mSAD4	cage_2_ex	80	4.35111254	0.89698438	7.18911116
mSAD5	cage_2_ex	88	4.23504497	0.89685054	6.88549864
mSAD6	cage_2_ex	99	4.6352924	0.90669609	8.04691229

Similarity metrics were determined via the ASV output table of QIIME2 (v2021.11), and chao1, Shannon, Simpson, and Faith –p-metric QIIME2 plug-ins. The table displays alpha diversity metrics for: chao1, Shannon, Simpson, and Faith_pd, clustered according to Cage column.

## Discussion

Obesity is a defining characteristic of metabolic syndrome and is primarily classified by increased weight gain and adiposity (39). mSAD-fed mice exhibited significantly higher weight gain and adiposity relative to CON mice, even though overall dry matter, protein, and calorie intake were similar. These data emphasize that the nutrient content of the diet, not necessarily the energy content, is important in controlling energy storage and allocation, leading to weight gain and alterations in other physiological outcomes associated with MetS. These data further suggest that specific nutrients or combinations of nutrients, primarily carbohydrates or fats that are typically associated with the induction of obesity and MetS in humans, are inducing obesity and MetS related co-morbidities in male C57BL/6J mice. Since protein intake was similar between the diet treatments, we suggest protein was not directly an effector of obesity and MetS. Consequently, human-relevant diets can be formulated for C57BL/6J mice to emulate the physiological outcomes associated with obesity and MetS observed in humans (2).

Previous studies using NHANES-based diets that reflect western consumption patterns further demonstrate the effect of diet quality on metabolic outcomes. The TWD, although similar to the mSAD, is different in its formulation and includes lower n-6: n-3 and saturated fat levels. Interestingly, average weekly calorie intakes of mSAD and TWD mice were relatively similar, but weight-related outcomes were dramatically different at the end of each study (3). Comparatively, the SAD contains n-6: n-3 and saturated fat levels more similar to the mSAD and induced weight gain in a similar manner to the mSAD (11). Intake of both saturated fat and omega-6 PUFAs have been associated with an increased risk for obesity (40). In the current study, mice fed the mSAD exhibited equivalent food intake to the AIN93M-fed controls in terms of total dry matter, total protein, and total energy, but differed substantially in intake of fat and carbohydrate quantity and quality, leading to an obese phenotype. We hypothesize that specific lipids or carbohydrates in the diet, or a complex ratio therein, affect body mass outcomes leading to a MetS phenotype. Furthermore, the fact that significant differences in specific growth rates were observed in the first week of feeding suggests that the metabolic triggers and cell signaling cascades associated with MetS phenotypes are induced rapidly and persist as long as the diet is consumed.

Glucose dysregulation and HFD are both associated with the development of type 2 diabetes (T2D) in rodent models and humans (41–43). Within 4 weeks of diet intake, mSAD-fed mice developed hyperglycemia, glucose intolerance, and reduced insulin sensitivity relative to CON mice. Increased adiposity in obese individuals is linked to elevated secretion of adipokines and pro-inflammatory cytokines, which may provide a mechanistic basis for the development of insulin resistance (44). A high fat/high carbohydrate diet has been associated with increased levels of TNFα and was shown to activate downstream pathways (45). Interestingly, TWD-fed C57BL/6J mice did not exhibit metabolic effects of glucose metabolism seen in mSAD-fed mice. In comparison, mice fed the SAD displayed fasting hyperglycemia and reduced glucose tolerance after 20 weeks of diet (11). Moreover, SAD-fed rats exhibit increased gene expression levels of pro-inflammatory mediator TNFα (46). Altogether, these data suggest that mSAD promotes a more rapid induction of hyperglycemia and insulin resistance as compared to TWD or the SAD.

A notable difference in the mSAD and SAD is the inclusion of HFCS as a replacement for sucrose. HFCS is found in numerous consumer products, and dietary exposure to sucrose or HFCS may produce different metabolic and physiological effects, even when controlled for energy intake (17). Previous studies have investigated the effects of HFCS-infused drinking water; however, HFCS was included as an ingredient in the mSAD and not supplemented in the water to limit the direct effects of palatability on water consumption. Sweetened water may promote the consumption of liquid calories in place of dietary calories, thereby limiting the intake of the diet (23). Fructose has been implicated in the development of T2D (14), NALFD (47), and gut dysbiosis (48); however, conflicting studies (18) highlight the importance of further investigation into the source of fructose as an underlying factor in the development of these disorders.

Analysis of overall fat distribution revealed significant increases in visceral gonadal adipose tissue (VAT) depots. Accumulation of VAT is strongly linked to the development of T2D (36, 49) and may provide further insight into metabolic alterations induced by mSAD feeding. Expansion of VAT depots in murine DIO models results in enhanced recruitment of macrophages to adipose tissue that exhibit a pro-inflammatory M1 phenotype when compared to controls (50). During advanced obesity, these macrophages manifest histologically as rings of F4/80+ cells surrounding adipocytes called crown-like structures, and nearly all adipose-tissue derived TNFα is produced by macrophages (51). TNFα is well known to directly decrease insulin sensitivity (52) through proinflammatory signaling via the NF-κβ and JNK pathways. We suggest the presence of CLS in fat depots concomitant with increased levels of TNFα in the colon are related to alterations in glucose metabolism in mSAD-fed mice.

The development of insulin resistance is thought to play a significant role in the progression of NAFLD, a common manifestation of MetS. A high-fat, low-carbohydrate diet promotes increasing levels of liver fat when compared to an isocaloric low-fat, high-carbohydrate diet (53). Diets high in saturated fat with low PUFA levels have also been shown to promote increases in liver fat (54). In the present study, two of six mSAD-fed mice displayed hepatic steatosis. Interestingly, one CON mouse exhibited a similar phenotype. Although the AIN-93M control diet is formulated to support basic health and reproduction of murine models, there is one reported instance of liver steatosis formation in response to AIN-93M consumption (55), indicating that consumption of AIN-93 may, in some cases, induce NAFLD. Consumption of the SAD diet has been reported to increase liver triglycerides; however, the presence of NAFLD was not evaluated (11, 46). Liver steatosis may be an outcome that develops over longer periods and additional studies that focus on human-relevant dietary induction of NAFLD are warranted.

Reports of gut dysbiosis associated with WD consumption have led to increased interest in the role of the microbiome in the development of obesity and MetS. Several studies have linked WD-induced pathologies to altered populations of microbes (1, 24, 56) and reversion of gut dysbiosis has been shown to attenuate WD-induced dysfunction (57). The limited amount of microbiome data reported from previous NHANES-based WD studies prompted us to investigate the composition of the fecal microbiome of mSAD-fed mice. The separation between control cage 1 and control cage 2 in the PCOA plot was of interest during the analyses. The two genera, *Romboutsia* and *Faecalibaculum* are shown to most likely be the primary components driving the separation of these cages. Interestingly, both of these genera of microbes belong the phylum Firmicutes and both have been identified in the gut of healthy patients and animals (58–61). Although these bacteria introduce variation between the control cages, we believe this variation does not represent an unhealthy population or that these animals have potential gut dysbiosis. Differences in microbial composition between diet groups were primarily of interest, as they may provide insight into physiological changes seen in mSAD mice. Similar to reported studies on diet-induced obesity, the fecal composition of mSAD mice was enriched with microbes from the Firmicutes phylum (62, 63). Increases in levels the Lachnospiraceae family were particularly interesting, as this family of microbes has been positively associated with multiple metabolic disorders, including alterations in glucose and lipid metabolism, onset of T2D, and diagnosis of NAFLD (64–68). Reduced levels of bacteria from the *Akkermansia* genus indicate a potential loss of protective microbes in the gut of mSAD mice. *Akkermansia mucinophilia*, the primary known species in this genus, is a mucin-degrading bacteria that has been documented to be altered under WD regimens (69). Levels of this species have shown to be inversely correlated with body weight in response to HFD (69) and reduced in models of type 2 diabetes, and administration of this species as a probiotic has shown to have protective effects on atherosclerosis, inflammation, and HFD-induced metabolic disorders (70, 71). Reduction of *Akkermansia* microbes in conjunction with altered glucose metabolism and increased body weight in mSAD mice agrees with existing reported data. The elevated presence of microbes from the *Ileibacterium* genus in the mSAD group may also be related to dietary intervention. This newly described genus was documented to be increased in response to HFD administration and positively correlated with serum lipid levels in a murine atherosclerosis model (72).

We recognize that microbial population dynamics observed alone cannot define the mechanism of action that resulted in increased weight gain, adiposity, and related co-morbidities. However, these trends lead us to hypothesize that shifts in microbial populations could result in a diet-dependent alteration of the metabolome. In fact, in a companion study, a metabolomic assessment of a Trp metabolite panel revealed diet-induced alterations in several Trp metabolites (24). Both nutrition and microbial populations are reported to be important in Trp metabolism and, as such, in maintaining systemic homeostasis including nutrient sensing, metabolic stress response, immunity (73), as well as neurological and behavioral interactions (24). Collectively, changes in diet, microbial populations and Trp metabolism could be important in the understanding progression of diet-induced metabolic disease and treatment options.

It is apparent that diet quality exerts pronounced effects on multiple physiological systems and signaling among these systems plays an integral role in the onset and development of metabolic disease (1, 41, 49, 74). The increased prevalence of obesity (75) and associated strain placed on the healthcare system (76) highlight the importance of developing research models that accurately reflect affected populations. Additionally, it is important to develop diets that initiate obesity and MetS to better understand the relation between diet and metabolic disease. Many previously established diets used to study DIO lacked relevant ingredients that reflect the consumption patterns that may ultimately induce and/or exacerbate metabolic disorders (1, 3, 77). The mSAD is formulated based upon the most recent data reflecting United States consumption patterns and induces pathologies consistent with MetS at a rate faster than SAD. We hypothesize the rapid development of MetS in C57BL/6J mice fed mSAD is due to either the inclusion of HFCS in the diets or interaction of carbohydrate and fat sources, leading to an aberrant metabolic cascade mediated by the gut-brain axis. We propose the mSAD diet induces the natural development of obesity-related pathologies and, therefore, becomes another important tool for improving rodent models of DIO and MetS. Moreover, this new diet can be reverse engineered to look for nutrient patterns and ratios contributing to disease outcomes, providing both a positive and negative control for future studies. This diet can also be used to address therapeutic targets and other interventions. We suggest that a new generation of diets can be developed in a variety of animal models, leading us to understand the basic universal cascades associated with consumption patterns leading to excessive weight gain and related co-morbidities.

## Data availability statement

The datasets presented in this study can be found in online repositories. The names of the repository/repositories and accession number(s) can be found below: https://www.ncbi.nlm.nih.gov/, PRJNA718626.

## Ethics statement

The animal study was reviewed and approved by Institutional Animal Care and Use Committee at the University of Alabama at Birmingham.

## Author contributions

SW, GK, JB, CM, DS, MP, and MM conceived of, designed, and supervised the study. SC, CG, AC, GG, BVa, AM, MW, TB, WV, and LW assisted with data collection and interpretation. SC, CG, GG, and WV assisted with statistical analyses, interpretation, and presentation under supervision of SW, CM, JB, DS, and GK. SC prepared initial versions of the manuscript with all authors providing input and final approval of the submitted and published versions. All authors contributed to the article and approved the submitted version.
