# Supplementary material for: A modified standard American diet induces physiological parameters associated with metabolic syndrome in C57BL/6J mice
Source: Front Nutr. 2022 Aug 29;9:929446. doi: 10.3389/fnut.2022.929446 (PMC9464921; doi:10.3389/fnut.2022.929446)
Supplement: Supplementary file 1 [file Data_Sheet_2.PDF]

Group 1	Group 2	Sample size	pseudo-F	p-value	q-value
cage_1_Con	cage_1_Ex	6	13.4677552	0.103	0.106
cage_1_Con	cage_2_Con	6	13.0365431	0.1	0.106
cage_1_Con	cage_2_Ex	6	20.9922115	0.095	0.106
cage_1_Ex	cage_2_Con	6	6.97750278	0.106	0.106
cage_1_Ex	cage_2_Ex	6	9.43339966	0.092	0.106
cage_2_Con	cage_2_Ex	6	15.5837506	0.092	0.106

Supplementary Table 1. PERMANOVA beta diversity metrics determined via "qiime diversity beta-group-significance" plug-in.

Df	SumsOfSqs	SumOfSqs	MeansSqs	F.model	R2	Pr(>F)
Cage_Effect	3	1.43219494	0.47739831	12.7195813	0.82668506	0.001
Residuals	8	0.30026039	0.03753255	NA	0.17331494	NA
Total	11	1.73245533	NA	NA	1	NA

Supplementary Table 2. Adonis beta-diversity metrics were determined via "qiime diversity beta-group-significance" plug-in.

	OTU_ID	C1	C2	C3	C4	C5	C6	mSAD1	mSAD2	mSAD3	mSAD4	mSAD5	mSAD6
1	Akkermansia	42.9	33	57.2	20	27.2	7.4	15.9	12.3	12.9	16.5	15.6	11.2
2	Ileibacterium	4.9	14.7	10	19.8	22.8	27.4	42.1	34.8	50.1	25	22.9	25.9
3	Lachnospiraceae	3.5	4.2	2.7	7	3.1	3.8	7	7.5	4.5	11.3	11.4	15.3
4	Faecalibaculum	0	0	0	11.9	22.6	31.5	2.5	0.5	4.8	0.7	0.5	0.7
5	Romboutsia	23.5	16.4	9.4	0	0	0	0	0	0	0	0	0
6	Lachnospiraceae	0.1	0.1	0.2	1.8	0.8	0.6	0.9	1.9	0.5	13.5	19	14.7
7	Muribaculaceae	0.2	0.8	0.7	11.3	4.2	5.3	3.2	4.9	3.1	3.4	2.7	2.8
8	Lactobacillus	4.9	7.2	1.6	2.7	1.1	1.7	0.3	0.3	0.6	1.9	3.6	2.1
9	Lachnospiraceae	0.6	0.3	0.8	0.5	0.4	0.7	0.7	0	0.3	7.1	8.7	9.5
10	Dubosiella	0.8	0.8	0.7	3.9	5.1	7.8	2.2	2.1	5.8	0	0.1	0
11	Lactococcus	4.1	2.9	3.1	1.3	0.4	0.5	0.2	0.4	0	0.6	0.4	0.4
12	Bacteroides	0.5	1.3	1.4	3.6	1.5	2.2	1.2	1.6	0.7	0.6	0.9	0.7
13	Blautia	0.6	0.6	0.8	4.5	2.7	1.7	1.4	0.9	0.9	0	0.4	0.1
14	Lachnoclostridium	0.8	1.1	0.8	0.2	0	0	2	6.6	1.3	0.5	0.6	0.9
15	Coriobacteriaceae	0.4	0.8	0.4	1.9	2.4	2.6	1	0.5	1.7	0.6	0.7	1
16	Clostridium	2	7.3	1.3	0.1	0	0	0	0	0.1	0	0	0
17	Helicobacter	0	0	0	0	0	0	9	0	0	0	3.7	0
18	Lachnospiraceae	0	0	0	0	0	0	4.5	2.2	0.5	3.6	1.7	1.9
19	Lachnospiraceae	0.6	0.5	0.3	0.1	0.1	0.1	1.2	0.2	0	2.8	2.1	2.7
20	Bifidobacterium	0	0	0	1.5	2	2.8	0.9	0.4	2.4	0.8	0.5	0.8

21	Streptococcus	0.2	0.5	0.6	0	0	0	0	3.7	0.7	1.7	0	1.8
22	Roseburia	0.3	0.4	0.4	0.3	0.1	0.1	0	2	1.3	0.4	0.5	0.6
23	Colidextribacter	0.2	0.4	0.6	1.1	0.9	0.6	0.2	0.3	0	0.2	0.2	0.3
24	Parasutterella	0.2	0.1	0.3	1.2	0.3	0.4	0.8	0.4	0.8	0.3	0.3	0.3
25	Prevotella	0.6	0.6	0.5	0	0	0	0	2.2	0.2	0.8	0	0.2
26	coprostanoligenes	0.3	0.6	0.4	0.8	0.1	0.2	0	0.3	0.1	0.5	0.3	0.3
27	Veillonella	0.5	0.3	0.1	0	0	0	0	1.6	0.5	1.2	0	0.3
28	xylanophilum	0.2	0	0.5	0	0	0	0.7	0.7	0.5	0.7	0.4	0.5
29	Clostridia_vadin	1.2	0.3	0.3	0.8	0.1	0.3	0	0	0	0	0	0
30	Alistipes	0.1	0.2	0.1	0	0	0	0.2	0.2	0.2	0.7	0.5	0.6
31	GCA-900066575	0	0.1	0	0.2	0.2	0.2	0.2	0.5	0.3	0.3	0.4	0.6
32	Lachnospiraceae	0	0.2	0.1	0.2	0.2	0.1	0.2	0.3	0.4	0.3	0.5	0.4
33	Pasteurellaceae	0	0	0.5	0	0	0	0	2	0.3	0.4	0	0.1
34	Anaeroplasma	1.7	0.1	0.3	0.1	0	0	0	0	0	0	0	0
35	Neisseria	0	0.2	1	0	0	0	0	1.2	0.2	0	0	0.1
36	Oscillibacter	0	0	0.1	0.7	0.5	0.3	0.2	0.2	0.2	0.1	0.1	0.3
37	Oscillospiraceae	0.2	0.3	0.3	0.5	0.2	0.2	0	0.2	0	0	0.1	0.2
38	Dorea	0	0	0	0	0	0	0.2	0.5	0.1	0.7	0.4	0.7
39	Comamonadaceae	0.4	0.4	0.1	0	0	0	0	0.7	0.9	0	0	0
40	Anaerotruncus	0	0	0	0.1	0	0	0.4	0.7	0.3	0.2	0.2	0.4
41	Erysipelatoclostridium	0.6	0.4	0.2	0.1	0	0	0.1	0.1	0	0	0	0
42	Oscillospiraceae	0	0.1	0	0.4	0.2	0.3	0.1	0.3	0	0	0	0.1
43	Actinobacillus	0	0.6	0	0	0	0	0	0.7	0	0	0	0.3
44	Incertae_Sedis	0.1	0.2	0.3	0.2	0.1	0	0	0.2	0.1	0.1	0	0.1
45	Ruminococcus	0	0	0	0	0	0	0.1	0.2	0.2	0.5	0.3	0.2
46	Acetatifactor	0	0	0	0.3	0.2	0.4	0.2	0	0.2	0	0	0
47	Fusobacterium	0	0	0	0	0	0	0	0.5	0	1.1	0	0
48	Lachnospiraceae	0.1	0.3	0.3	0.2	0	0	0	0	0	0	0	0.1
49	RF39	0.2	0.1	0.1	0	0	0	0	0.2	0.1	0.1	0	0.2
50	Sphingomonas	0.2	0.2	0	0	0	0	0	0.3	0.4	0	0	0
51	Christensenellaceae	0.2	0.1	0.2	0	0.1	0.1	0	0	0	0	0	0
52	UCG-005	0.1	0.2	0.1	0.4	0	0	0	0	0	0	0	0
53	Anaerovoracaceae	0.3	0.2	0.1	0.1	0	0	0	0	0	0	0	0
54	Ruminococcaceae	0	0	0	0.2	0.2	0.2	0	0	0	0	0.1	0
55	Alloprevotella	0	0	0.1	0	0	0	0	0.3	0.7	0	0	0
56	Gemella	0	0.1	0.3	0	0	0	0	0.2	0	0	0	0
57	Herbaspirillum	0.1	0.1	0	0	0	0	0	0.3	0.3	0	0	0
58	Enterococcus	0.4	0	0.1	0	0	0	0	0	0	0	0	0
59	Bacilli	0	0	0	0	0	0	0	0	0	0	0	0.5
60	Intestinimonas	0	0	0	0.1	0	0	0.1	0	0	0.1	0.1	0.1
61	Acinetobacter	0.1	0	0	0	0	0	0	0.3	0.2	0	0	0

62	Clostridia	0.1	0.1	0.1	0	0	0	0	0.1	0	0	0	0
63	Granulicatella	0	0	0	0	0	0	0	0.6	0	0	0	0
64	Peptococcaceae	0	0	0	0.1	0.1	0	0	0	0	0.1	0.1	0.1
65	Rothia	0	0.1	0	0	0	0	0	0	0	0.2	0	0.1
66	Megasphaera	0.2	0	0	0	0	0	0	0.1	0	0.1	0	0
67	Small_Abundance	0	0	0.3	0	0	0	0	0	0	0	0	0
68	Small_Abundance	0	0	0	0	0	0	0	0.3	0	0.1	0	0
69	Small_Abundance	0.2	0	0	0	0	0	0	0	0	0	0	0
70	Small_Abundance	0	0	0	0	0	0	0	0	0.4	0	0	0
71	Small_Abundance	0.1	0	0	0	0	0	0	0	0.1	0	0	0
72	Small_Abundance	0.1	0	0	0	0	0	0	0.2	0	0	0	0
73	Small_Abundance	0.1	0	0.1	0	0	0	0	0	0	0	0	0
74	Small_Abundance	0.1	0	0	0	0	0	0	0	0	0	0	0
75	Small_Abundance	0.1	0	0	0	0	0	0	0.1	0	0	0	0
76	Small_Abundance	0	0	0	0	0	0.1	0	0	0	0	0	0
77	Small_Abundance	0	0	0.1	0	0	0	0	0	0	0	0	0
78	Small_Abundance	0	0	0	0	0	0	0	0	0	0	0	0.1
79	Small_Abundance	0	0	0	0	0	0	0	0	0	0.1	0	0
80	Small_Abundance	0	0.1	0	0	0	0	0	0	0	0	0	0
81	Small_Abundance	0	0	0	0	0	0	0.1	0	0	0	0	0
82	Small_Abundance	0	0	0	0.1	0	0	0	0	0	0	0	0
83	Small_Abundance	0	0	0	0	0	0	0	0.1	0	0	0	0
84	Small_Abundance	0	0	0	0	0	0	0	0	0	0	0	0
85	Small_Abundance	0	0	0	0	0	0	0	0.1	0	0	0	0
86	Small_Abundance	0	0	0	0	0	0	0	0	0	0.1	0	0
87	Small_Abundance	0	0.1	0	0	0	0	0	0	0	0	0	0
88	Small_Abundance	0	0	0	0	0	0	0	0	0	0	0	0
89	Small_Abundance	0	0	0	0	0	0	0	0	0	0	0	0
90	Small_Abundance	0	0	0	0	0	0	0	0.1	0	0	0	0
91	Small_Abundance	0	0	0	0	0	0	0	0.1	0	0	0	0
92	Small_Abundance	0	0	0	0	0	0	0	0.1	0	0	0	0
93	Small_Abundance	0	0	0	0	0	0	0	0	0	0	0	0
94	Small_Abundance	0	0	0	0	0	0	0	0	0	0	0	0
95	Small_Abundance	0	0	0	0	0	0	0	0	0	0	0	0
96	Small_Abundance	0	0	0	0	0	0	0	0	0	0	0	0
97	Small_Abundance	0	0	0	0	0	0	0	0	0.1	0	0	0
98	Small_Abundance	0	0	0	0	0	0	0	0	0	0	0	0
99	Small_Abundance	0	0	0	0	0	0	0	0	0	0	0	0
100	Small_Abundance	0	0	0	0	0	0	0	0	0	0	0	0

Supplementary Table 3. Percent relative abundance showing top 100 taxa at the most resolved level across all samples (n = 3 for each sample) in the gut ecosystem of *mus musculus*. Taxonomic identities were based on their assignment through the (SILVA v138) database as determined by the Quantitative Insights into Microbial Ecology (QIIME_2, v2021.11) and graphed using R (ggplot package).

sample-id	input	filtered	percentage of input passed filter	denoised	merged	percentage of input merged	non-chimeric	percentage of input non-chimeric	Shannon	Simpson
C1	136071	52030	38.24	51197	39198	28.81	37475	27.54	3.41916094	0.77558052
C2	153383	49203	32.08	48327	37362	24.36	36050	23.5	3.90299357	0.84905891
C3	118602	42571	35.89	41869	35617	30.03	35313	29.77	2.96225955	0.65589128
C4	161567	42446	26.27	41645	35143	21.75	32909	20.37	4.24052121	0.90009793
C5	131817	40402	30.65	39796	34474	26.15	30456	23.1	3.31761141	0.82603949
C6	157397	39047	24.81	38490	30404	19.32	25872	16.44	3.44943346	0.82690652
mSAD1	127691	35551	27.84	34838	25620	20.06	25312	19.82	3.68744323	0.82162558
mSAD2	126987	33434	26.33	32582	24647	19.41	24383	19.2	4.65994564	0.88047728
mSAD3	86461	22568	26.1	21979	15667	18.12	15411	17.82	3.4966321	0.77587981
mSAD4	78594	23689	30.14	23190	19623	24.97	19562	24.89	4.35111254	0.89698438
mSAD5	184777	52997	28.68	52398	44022	23.82	42580	23.04	4.23504497	0.89685054
mSAD6	167869	45922	27.36	45117	35071	20.89	31824	18.96	4.6352924	0.90669609

Supplementary Table 4. Denoising statistics for quality filtering performed using DADA2 (q2-dada2).
